# The Price of Homemade Street Food in Central Asia and Eastern Europe: Is There a Relation with Its Nutritional Value?

**DOI:** 10.3390/foods10091985

**Published:** 2021-08-25

**Authors:** Gabriela Albuquerque, Sofia Sousa, Marcello Gelormini, Inês Lança de Morais, Susana Casal, Olívia Pinho, Albertino Damasceno, Pedro Moreira, João Breda, Nuno Lunet, Patrícia Padrão

**Affiliations:** 1 EPIUnit—Instituto de Saúde Pública, Universidade do Porto, 4050-600 Porto, Portugal; sofia.sousa@ispup.up.pt (S.S.); sucasal@ff.up.pt (S.C.); tino_7117@hotmail.com (A.D.); pedromoreira@fcna.up.pt (P.M.); nlunet@med.up.pt (N.L.); patriciapadrao@fcna.up.pt (P.P.); 2 Laboratório para a Investigação Integrativa e Translacional em Saúde Populacional (ITR), 4050-600 Porto, Portugal; 3 Faculdade de Ciências da Nutrição e Alimentação da Universidade do Porto, 4150-180 Porto, Portugal; oliviapinho@fcna.up.pt; 4 Division of Noncommunicable Diseases and Life-Course, World Health Organization (WHO) Regional Office for Europe, DK-2100 Copenhagen, Denmark; marcello.gelormini@gmail.com (M.G.); inesbolm@gmail.com (I.L.d.M.); 5 LAQV/REQUIMTE, Laboratório de Bromatologia e Hidrologia, Faculdade de Farmácia, Universidade do Porto, 4050-313 Porto, Portugal; 6 Departamento de Ciências da Saúde Pública e Forenses e Educação Médica, Faculdade de Medicina da Universidade do Porto, 4200-319 Porto, Portugal; 7 Faculdade de Medicina, Universidade Eduardo Mondlane, Maputo 254, Mozambique; 8 Centro de Investigação em Atividade Física, Saúde e Lazer, Universidade do Porto, 4200-450 Porto, Portugal; 9 WHO Regional Office for Europe, 10675 Athens, Greece; rodriguesdasilvabred@who.int

**Keywords:** Central Asia, Eastern Europe, food price, nutritional value, ready prepared foods, street food

## Abstract

The aim was to describe the price of homemade street foods in four cities of Central Asia and Eastern Europe and to analyze its association with energy density, macronutrients, sodium and potassium contents. Cross-sectional evaluations of street food vending sites were conducted in Dushanbe, Tajikistan (*n* = 562), Bishkek, Kyrgyzstan (*n* = 384), Almaty, Kazakhstan (*n* = 236) and Chișinău, Moldova (*n* = 89) in 2016–2017. Information on the homemade street foods available, including price, was systematically collected; the most commonly available foods (*n* = 64) were sampled for chemical analysis. Prices were converted to international dollars ($) and expressed as $/serving, $/100 g and $/100 kcal. The median street food price was $1.00/serving, $0.85/100 g and $0.33/100 kcal. Traditional foods were cheaper per 100 g than the westernized ($0.77 vs. $1.00, *p* = 0.011). For each unit increase in energy density (kcal/g), the homemade street foods were $0.12 cheaper per 100 kcal and $0.11 more expensive per 100 g. The carbohydrate content was negatively associated with price per 100 kcal, while total fat, monounsaturated, saturated and trans-fatty acids content were positively associated with price per 100 g. Energy-dense homemade street foods were the cheapest. Further insight on food preparation might clarify the association between carbohydrates and monounsaturated fatty acids content and street food price.

## 1. Introduction

Food price is a well-established determinant of food choice and diet quality. Nutrient-dense foods are globally more expensive than energy-dense foods, with the latter generally being less healthy [1,2,3,4,5]. This observation is especially relevant in low-income populations who face budget constraints and make food choices to meet daily energy needs at the lowest cost [2,6]. The greater affordability of energy-dense foods has been associated with the high burden of obesity and non-communicable diseases (NCDs) in high- [6] and low–middle-income countries (LMICs) undergoing the nutrition transition [3,7]. The Food and Agriculture Organization (FAO) recently reported the coexistence of undernutrition, micronutrient deficiencies, and diet-related NCDs in Central Asia and Eastern Europe, signalling this as a marker of poor diets and limited access to nutritious diets due to the cost of food [8].

Street food is highly relevant for the diet of many families in LMICs, often replacing home meals [9]. In Central Asia and Eastern Europe, street food is part of the food culture, reflecting the availability of local products, culinary practices, lifestyles and consumer preferences [10]. Low cost, along with convenience, taste, and considerable portion sizes, are factors that are frequently prioritised over nutritional value [9,10]. Despite the currently growing knowledge on its nutritional characteristics, it has been reported that street food is a meaningful source of fat, namely saturated (SFA) and *trans*-fatty acids (TFA), salt and sugar [9], particularly when considering homemade street food products in the region [11,12,13]. According to studies conducted in urban environments in Central Asia and Eastern Europe, the most frequently available street foods are homemade, mostly cooked and presented in large servings. The most popular food groups were main dishes, sandwiches and savoury pastries, of which a great proportion is meat-based, and bread, snacks, sweet pastries, cakes and cookies [11,12,13].

Current evidence on the association between food price and nutritional composition has been mostly drawn from research in high-income countries [1,2]. Therefore, this study aimed to describe the price of homemade street foods in four cities of Central Asia and Eastern Europe (Dushanbe, Tajikistan; Bishkek, Kyrgyzstan; Almaty, Kazakhstan; Chișinău, Moldova) and to analyse its association with their nutritional composition (energy density, protein, carbohydrates, lipids, fatty acids profile, sodium and potassium contents).

## 2. Materials and Methods

This study was based on four cross-sectional evaluations implemented as part of the FEEDcities project, supported by the World Health Organization (WHO)—Europe, which used a stepwise standardised framework to characterise the street food environment in urban areas of Central Asia and Eastern Europe [14]. Data from four cities/countries were included; the first evaluations were conducted between May and August 2016 in Dushanbe (Tajikistan), Bishkek (Kyrgyzstan) and Chișinău (Moldova), and an additional assessment was conducted between July and August 2017 in Almaty (Kazakhstan).

**Figure 1:**
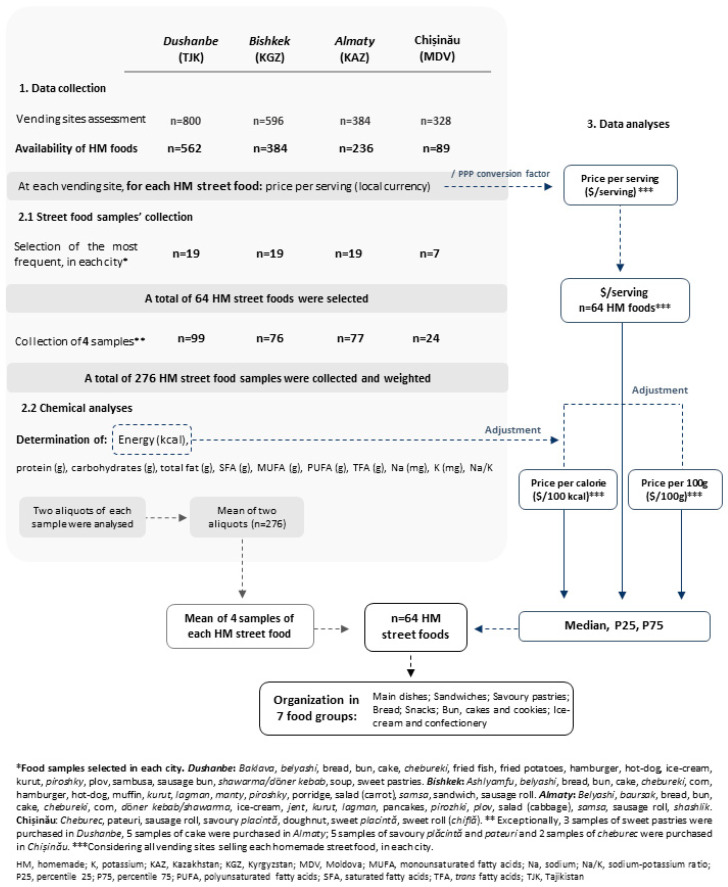
Flowchart of the sampling procedure, data and food samples’ collection and analyses, conducted in Dushanbe (Tajikistan), Bishkek (Kyrgyzstan), Almaty (Kazakhstan) and Chișinău (Moldova).

Figure 1 illustrates the methodological procedures involved in this study, including sampling, data and food samples’ collection, and analyses.

### 2.1. Eligibility Criteria

The definition proposed by the FAO and the WHO for street food was used—“ready-to-eat foods and beverages prepared and/or sold by vendors or hawkers especially in the streets and other similar places” [15,16]. Eligible vending sites were defined as the business establishments selling ready-to-eat food, including beverages and/or snacks, from any venue other than permanent storefront businesses or establishments with four permanent walls which did not sell directly to the street. Mobile vendors and sellers with semi-static or stationary vending units were included. The exclusion criteria were as following: (1) food establishments with four permanent walls; (2) permanent storefront business; (3) street vendors exclusively selling non-food products or raw foods which were not ready-to-eat; (4) food stalls and carts that were part of permanent stores or licensed establishments.

Industrial street foods (commercially prepared foods sold by street vendors without further preparation and/or cooking) [10] were not considered in analyses; only homemade street foods (self-prepared foods, at home or on the street, even if using industrial ingredients) were addressed in this study.

### 2.2. Sampling of Markets and Vending Sites

The street food vending sites in these four cities were located inside or in the proximity of urban markets [11,12,13]. The sampling strategy began with identifying all public markets (Dushanbe, *n* = 36; Bishkek, *n* = 19; Almaty, *n* = 24; Chișinău, *n* = 1). A total of 10 markets were randomly selected in each Central Asian city, and the only central market in Chișinău, Moldova was included. Afterwards, a buffer was drawn around the centroid of each market with a 500 m diameter in Dushanbe, Bishkek and Almaty, and a 1 km diameter in Chișinăuto define the study area.

Trained interviewers canvassed all publicly accessible streets to identify all eligible street food vending sites in each study area. In the Central Asian cities, the selection of the vending sites to be assessed was based on the estimated number of vending sites in each market [14]. In markets with 100 or fewer vending sites (all markets in Dushanbe and eight markets in Almaty), the vendors from all eligible vending sites were invited to participate. In markets with more than 100 vending sites (all markets in Bishkek and two markets in Almaty), all vending sites were mapped with one in every two being systematically assessed, and the vendors were invited to participate. In Chișinău, all the vendors from the eligible vending sites were invited to participate.

### 2.3. Data Collection

The interviewers registered the Global Positioning System (GPS) coordinates of each vending site [14]. Afterwards, they approached the vendors to explain the study objectives and procedures and to ask for expressing consent to participate in the study. When the vendors agreed, they carried out a computer-assisted personal interview, enquiring about the foods available. For each homemade street food identified, the interviewers registered the price in local currency and the serving size in grams.

Street foods were classified according to the preparation method, as cooked or uncooked, and according to the origin of the culinary recipe as traditional (foods consumed locally and prepared using local ingredients and methods transmitted across generations, being part of the national folklore) or westernised (foods originally from Western countries but currently consumed on a global scale) [7]. Finally, foods were grouped based on the WHO nutrient profile model [17]: (1) main dishes; (2) sandwiches; (3) savoury pastries; (4) bread; (5) snacks; (6) buns, cakes and cookies; (7) ice-cream and confectionery.

A total of 800, 596, 384 and 328 eligible street food vendors agreed to participate in Dushanbe, Bishkek, Almaty and Chișinău, respectively (participation ranged from 74.7% in Chișinău to 98.7% in Bishkek). Of these, 562 (70.2%), 384 (64.4%), 236 (61.4%) and 89 (27.1%) sold homemade street foods in Dushanbe, Bishkek, Almaty and Chișinău, respectively, and were therefore included in the present study (Figure 1).

### 2.4. Food Sample Collection

The most commonly available homemade street foods (19 in Dushanbe, Bishkek and Almaty each and 7 in Chișinău) were identified and collected for nutritional composition assessment.

The vending sites for food collection were randomly selected from the list of GPS coordinates of the eligible vending sites previously assessed (10 GPS coordinates at the location of 10 vending sites, which were randomly selected in each market). A sample of each food product, corresponding to one serving, was bought whenever possible at each of these vending sites. If not possible, a systematic selection procedure was followed, until vending sites where these foods were available were reached [14]. The street food samples were collected over 10 (Dushanbe and Bishkek), seven (Chișinău) and five (Almaty) consecutive days.

For most foods, four samples of all selected homemade street foods were collected from different vending sites. Exceptionally, three samples of sweet pastries were purchased in Dushanbe and five samples of cake were purchased in Almaty, while five samples of savoury plăcintă and pateuri and two samples of cheburec (traditional savoury pastries) were purchased in Chișinău. A total of 276 homemade street food samples were collected (Dushanbe, *n* = 99; Bishkek, *n* = 76; Almaty, *n* = 77; Chișinău, *n* = 24; Figure 1).

### 2.5. Chemical Analyses

Following collection, samples were homogenised, weighted and stored in a freezer (−18 °C) until the nutritional composition assessment, which included the following analysis: (a) moisture by oven drying at 103 °C until constant weight; (b) protein by Kjedahl’s method using a conversion factor of 6.25; (c) fat by Soxhlet’s method, (d) total minerals by dry ashing at 500 °C; (e) total carbohydrates plus fibre by calculating the difference. Further, energy values were estimated using the Atwater general factors (protein: 4 kcal/g; carbohydrates: 4 kcal/g; fat: 9 kcal/g). All procedures followed standard methods recommended by the Association of Analytical Communities (AOAC) [18]. The fatty acid composition (SFA, monounsaturated fatty acid (MUFA), polyunsaturated fatty acid s(PUFA), *n*-3 and *n*-6 fatty acids and TFA) analysis was performed by gas chromatography for fatty acids methyl esters. Sodium and potassium were analysed following a validated flame photometric method [19]. The analytical results were the average of two determinations per food sample. A third determination was conducted, if the coefficients of variation were above 5% for macronutrients and 1% for micronutrients. In this case, the average of the two determinations consistent with these criteria was calculated. All analytical results were expressed by serving size (the weight of each sample), in g. Furthermore, the serving and nutritional composition of each of the selected homemade street foods (*n* = 64) was computed as the mean of the samples of the same food item collected in each country (Figure 1).

### 2.6. Food Prices

The local currency prices of homemade street foods (price per serving) were converted to international dollars using purchasing power parity (PPP) exchange rates for household consumption. This indicator, retrieved from the World Bank, measures the amount of local currency units required to buy the same amount of goods and services in the domestic market as the US dollar would buy in the United States. For this study, the PPP exchange rates used were those extrapolated for 2016 (Tajikistan, Kyrgyzstan and Moldova) and 2017 (Kazakhstan) from the 2011 International Comparison Program (ICP) benchmark estimates [20]. Hereafter, “international dollar” was referred to as dollar ($).

Three food price metrics were defined: price per serving ($/serving), which was converted to price per 100 g of food ($/100 g) and price per calorie ($/100 kcal) [21]. To compute the price per calorie, the price of one serving was multiplied by 100 and divided by the energy in one serving (Figure 1).

### 2.7. Statistical Analyses

Median and percentiles (P25 and P75) were used to summarise street food prices ($/serving, $/100 g and $/100 kcal) according to the cultural origin of the recipe, preparation method and food group. Differences were tested using Kruskal–Wallis and Mann–Whitney U tests (Table 1). Median, P25 and P75 were also used to summarise the nutritional composition (energy (kcal), TFA (g), sodium (mg), potassium (mg) and molar sodium-to-potassium (Na/K) ratio) and price of each group of homemade street foods by country (Table 2).

**Figure 2:**
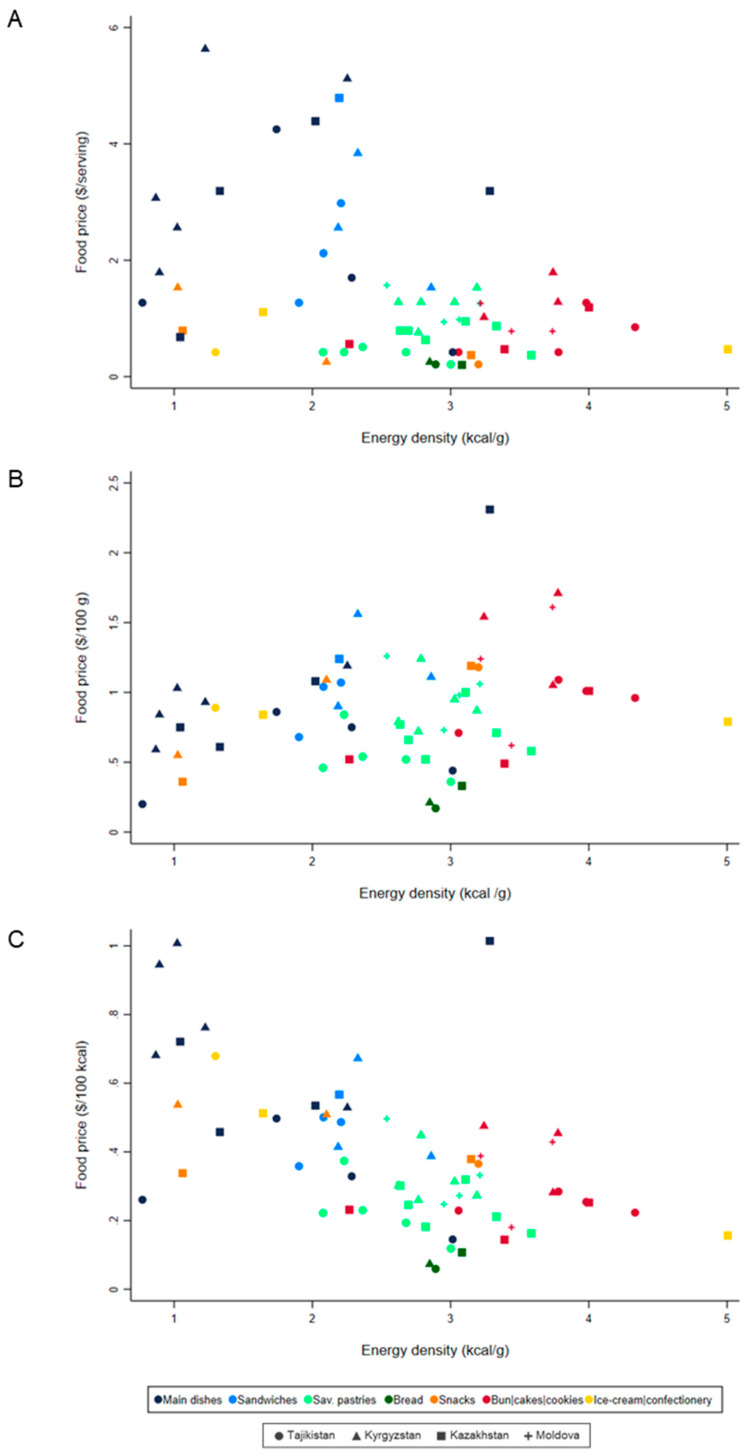
Relation between mean energy density (kcal/g) and median street food price, according to three different price metrics. (**A**) Price ($) per serving (β = −0.517; *p* = 0.002), (**B**) price ($) per 100 g (β = 0.112; *p* = 0.025), (**C**) price ($) per 100 kcal (β = −0.123; *p* < 0.001).

Scatterplots were used to represent the relation between energy density (kcal/g) and median price of homemade street foods ($/serving, $/100 g and $/100 kcal). Random effects linear regression models were used to quantify the relation between energy density (Figure 2) and between the nutrient content (protein, carbohydrates, total fat, PUFA, MUFA, TFA, SFA, sodium and potassium) (Table 3) and the median price of street foods. The coefficients presented in Table 3 for the relation between each nutrient and price/100 g were adjusted for energy contents, and those for the relation with price/100 kcal were adjusted for serving size.

A *p*-value (*p*) <0.05 was considered statistically significant. Statistical analyses were performed using the software STATA^®^, version 15.1 (StataCorp., College Station, TX, USA).

## 3. Results

### 3.1. Street Food Price

The median prices of homemade street foods was $1.00 per serving, $0.85 per 100 g and $0.33 per calorie. Westernised foods were significantly more expensive per 100 g than traditional ones (median price/100 g: $1.02 vs. $0.77, *p* = 0.011), while uncooked foods were significantly more expensive per energy unit than cooked foods (median price/100 kcal: $0.51 vs. $0.30, *p* = 0.006). A significant price variation by food group was also observed (price/serving: *p* < 0.001; price/100 g: *p* = 0.021; price/100 kcal: *p* < 0.001). Bread presented the cheapest median prices per serving (ranging from $0.20 in Kazakhstan to $0.25 in Kyrgyzstan), per 100 g (ranging from $0.17 in Tajikistan to $0.33 in Kazakhstan) and per calorie (ranging from $0.06 in Tajikistan to $0.11 in Kazakhstan). A low price per serving was also observed for snacks (ranging from $0.21 in Tajikistan to $0.89 in Kyrgyzstan), as well as a low price per calorie for savoury pastries (ranging from $0.22 in Kazakhstan to $0.38 in Moldova) and buns, cakes and cookies (ranging from $0.23 in Kazakhstan to $0.42 in Kyrgyzstan). Sandwiches had the highest median prices per serving (ranging from $2.12 in Tajikistan to $4.79 in Kazakhstan), and main dishes had the highest median prices per calorie (ranging from $0.35 in Tajikistan to $0.78 in Kyrgyzstan) (Table 1 and Table 2).

### 3.2. Street Food Nutritional Composition

Main dishes and sandwiches presented the highest median serving sizes (ranging from 217 g in sandwiches in Tajikistan to 395 g in main dishes in Kyrgyzstan), and energy per serving (ranging from 413 kcal in main dishes in Kyrgyzstan to 887 kcal in sandwiches in Kazakhstan). Snacks had the lowest median serving size and energy per serving (ranging from 16 g and 56 kcal/serving in Tajikistan to 116 g and 144 kcal/serving in Kazakhstan). TFA content varied from 0.00 g/serving (bread in Tajikistan) to 0.48 g/serving (sandwiches in Kyrgyzstan). Additionally, the median sodium content varied from 27 mg/serving (ice-cream and confectionery in Kazakhstan) to 1745 mg/serving (sandwiches in Kazakhstan), and the median potassium content varied from 36 mg/serving (snacks in Tajikistan) to 1284 mg/serving (sandwiches in Kazakhstan). The median Na/K ratio varied from 0 (ice-cream and confectionery in Kazakhstan) to 18 (snacks in Tajikistan; Table 2).

### 3.3. Association between Street Food Price and Nutritional Composition

#### 3.3.1. Price and Energy Density

For each unit increase in energy density (kcal/g), homemade street foods were $0.123 (*p* < 0.001) cheaper per 100 kcal and $0.112 (*p* = 0.025) more expensive per 100 g. Foods categorised as low-energy-dense (<2.0 kcal/g), main dishes as well as some snacks and sandwiches, were the most expensive per 100 kcal and the cheapest per 100 g, contrasting with buns, cookies and cakes, medium-to-high energy-dense, which were the cheapest street foods per 100 kcal and the most expensive per 100 g (Figure 2).

#### 3.3.2. Price and Macronutrients, Sodium and Potassium

A significant negative association was observed between carbohydrate content and price per 100 kcal (β = −0.0027; *p* = 0.020), after adjustment for serving size. In contrast, a significant positive association was observed between total fat (β = 0.0106; *p* = 0.028), MUFA (β = 0.0366; *p* = 0.016), SFA (β = 0.0218; *p* = 0.035) and TFA (β = 0.1504; *p* = 0.029) and food price per 100 g (Table 3).

## 4. Discussion

The median price of homemade street foods in urban areas of Central Asia and Eastern Europe was $1.00 per serving, with traditional homemade street foods being cheaper per 100 g than westernised options. Energy density was negatively associated with price per calorie and positively associated with price per 100 g. Moreover, carbohydrates were negatively associated, while total fat, MUFA, SFA and TFA were positively associated with food price.

The highest prices per calorie were found in low energy-dense traditional main dishes, vegetable and meat-based (porridge, salad, *lagman*, *ashlyamfu* and *plov*), sandwiches (hamburger, *shawarma* and hot-dog) and boiled corn. These street foods were among the cheapest, per 100 g, compared with, for example, energy-dense savoury pastries and buns, cakes and cookies. This may be justified by the higher water content of boiled corn, and vegetable-based main dishes and sandwiches, contributing to food weight but not providing energy or other nutrients [2]. Our findings are in line with a study on street food in South Africa [22] and with observations that fruits, vegetables and meat, especially lean, are more expensive per calorie, while refined grains, fats and sweets are cheaper [2,4,5,21,23]. Estimates from 2016 to 2017 in Tajikistan showed that fresh and micronutrient-rich foods were more expensive than staple foods [24]. Hence, it seems likely that ingredients such as meat, eggs, legumes, vegetables and nuts may have contributed to increase the price of these homemade street foods, due to their perishability and higher acquisition cost for vendors [3,5].

Bread is one of the cheapest staple foods in Central Asia [24]. In this study, including street foods collected in Central Asia and Eastern Europe, it was the cheapest group of street foods (regardless of the metric), which advocates for its promotion as an economic and healthy option for a snack, especially if whole grains-based. Other snack alternatives, such as cakes, cookies, confectionery and savoury pastries, were also cheap but had an expectedly higher energy density and a lower nutritional value. Furthermore, a negative association was observed between carbohydrates and food price. Previous studies have found similar associations, especially with refined grains and added sugars [3,21,25,26], which seems also to justify the lower prices of these street foods. In contrast, main dishes and sandwiches, which are likely to contain a considerable amount of starch and fibre, were among the most expensive per 100 kcal. In fact, complex carbohydrates have been previously associated with higher food prices, particularly in LMICs [3,25,26]. Thus, disentangling the proportion of starch, sugar and fibre within the carbohydrates construct might provide further insightful evidence concerning their possible differential contribution to the price of these street foods.

In the present study, a higher fat content was associated with a higher price per 100 g, in contrast with the conventionally described inverse association between fat content and lower food prices [2], but in line with findings from Iran [26]. However, the referred study results were expressed as correlations, which limit comparability with the current findings. The study from Iran [26] observed that MUFA and PUFA were positively (and weakly) correlated with food price; the results for total fat and SFA were not significant but showed a positive trend. In the current study, the association between fat content and food prices seemed to be in part explained by the MUFA content, which is present in diverse plant- (e.g., vegetable oils and nuts) and animal-based foods (e.g., red meats and high-fat dairies). As previously discussed, these foods are usually more expensive, which contributes to explaining these findings. A wide variability in MUFA content is found within most food groups, especially in savoury pastries, main dishes and sandwiches, which account for a large proportion of the street foods analysed. The different quantities or qualities of the ingredients used in their preparation by different vendors might explain this disparity [9,27]. This is expected to influence consumers’ health, as for example, while plant-based sources of MUFA may be beneficial, animal sources are recommended less often [28]. As such, further information about the ingredients used in the preparation of these homemade street foods might further elucidate the main sources of MUFA. Such evidence will potentially contribute to prioritising needed interventions to improve the nutritional composition [11,12,13] and prices of homemade street foods in this region. Examples include educational programmes for vendors promoting the replacement of some ingredients by healthier alternatives, along with policies to reduce the cost of healthy ingredients (e.g., oils and fats) for vendors, ensuring a lower final street food price.

The results of this study are relevant in the scope of the food insecurity burden in Eastern Europe and Central Asia. A considerable share of the household budget is spent on food (40% on average, ranging from 39.2% in Moldova to 60.7% in Tajikistan) [29]. Moreover, 2.4% of the population in Eastern Europe and 22.5% in Central Asia cannot afford a healthy diet [5]. This may underline, among more deprived households, a preference for cheaper options, disregarding nutrient-dense foods, such as fruits and vegetables [6]. This results in a significant proportion of the population with an energy-dense and nutrient-poor diet [8]. In Tajikistan, the daily diet of an average adult to only meet energy needs cost approximately $4.64 in 2016–2017, while a “lowest cost nutritious diet” would cost 2.5–3.5 times more [24]. By extrapolating the estimated per calorie price of street food ($0.33) for the recommended daily energy of 2000 kcal for an average adult, a daily diet based on street food would cost $6.60. Despite objectively uncertain, the contribution of street food to the daily diet of these populations is expectably high, given its wide availability [11,12]. Although it is unexpected to constitute the only source of food every day, these simple calculations show that the estimated daily cost of homemade street food seems to be closer to the average cost of a diet meeting only energy needs than to the “lowest cost nutritious diet”. Thus, ensuring access to healthier street foods at affordable prices seems to be a public health priority. Promoting traditional foods might be a possible strategy, given that these are cheaper than westernised options. In addition to the lower price, many of the traditional options are nutritionally interesting, given their vegetable and legume contents.

The stepwise systematic data collection of the FEEDcities study [14] allowed for adaptation to four different cities and the comparison of results between them, overcoming limitations of previous studies on street food [9,30]. Another strength is the assessment of the nutritional composition by chemical analysis, using reliable methodologies [9]. In this specific study, the conversion of food prices to international dollars based on PPP and inflation accounted for monetary value fluctuations across countries during the 2016–2017 period. The use of the median price contributed to minimising the price variability of a given food from one vending site to another. However, the limited number of foods within some food groups may have accounted for some lack of statistical power. Moreover, the use of random effects linear regression models to estimate the relation between nutrient content and food price aimed to account for unobserved heterogeneity between countries.

## 5. Conclusions

This study highlights a need to explore the contribution of street food to the diet of urban populations in Central Asia and Eastern Europe. Recommendations include the detailed assessment of the nutritional composition and the collection of robust data on ingredients and preparation methods. Additionally, the price comparison with industrial street foods and foods from different food outlets would contribute to contextualising street food in the scope of the food environment. Overall, this information might support sustainable policy action to create healthier and affordable urban food environments in the region.

## Figures and Tables

**Table 1:** Prices of street food by country and food characteristics, according to three metrics: price per serving, per 100 g and per 100 kcal.

	N	Median Street Food Price (P25–P75)					
		Price ($)/Serving		Price ($)/100 g		Price ($)/100 kcal	
Country							
Tajikistan	19	0.42	(0.42–1.27)	0.75	(0.46–1.01)	0.26	(0.22–0.37)
Kyrgyzstan	19	1.53	(1.28–2.56)	0.95	(0.79–1.19)	0.45	(0.30–0.67)
Moldova	7	0.98	(0.78–1.26)	1.06	(0.73–1.26)	0.33	(0.25–0.43)
Kazakhstan	19	0.79	(0.47–1.19)	0.75	(0.52–1.01)	0.30	(0.18–0.51)
		*p* = 0.016 *		*p* = 0.033 *		*p* = 0.041 *	
Origin of the culinary recipe							
Traditional	46	0.91	(0.42–1.53)	0.77	(0.54–1.03)	0.29	(0.21–0.50)
Westernised	18	1.27	(0.78–1.70)	1.02	(0.84–1.24)	0.40	(0.30–0.50)
		*p* = 0.266		*p* = 0.011 †		*p* = 0.092 †	
Preparation method							
Cooked	56	1.11	(0.53–1.75)	0.81	(0.56–1.05)	0.30	(0.23–0.47)
Uncooked	8	0.55	(0.31–1.32)	0.99	(0.84–1.14)	0.51	(0.38–0.70)
		*p* = 0.102		*p* = 0.141		*p* = 0.006 †	
Food group							
Savoury pastries	20	0.83	(0.46–1.27)	0.75	(0.56–0.97)	0.27	(0.22–0.32)
Buns|cakes|cookies	13	0.85	(0.56–1.26)	1.01	(0.71–1.24)	0.25	(0.23–0.39)
Main dishes	13	3.07	(1.70–4.25)	0.84	(0.61–1.03)	0.53	(0.46–0.76)
Sandwiches	7	2.56	(1.53–3.84)	1.07	(0.90–1.24)	0.49	(0.39–0.57)
Snacks	5	0.37	(0.25–0.79)	1.09	(0.55–1.18)	0.38	(0.37–0.51)
Bread	3	0.21	(0.20–0.25)	0.21	(0.17–0.33)	0.07	(0.06–0.11)
Ice-cream|confectionery	3	0.47	(0.42–1.11)	0.84	(0.79–0.89)	0.51	(0.16–0.68)
		*p* < 0.001 *		*p* = 0.021 *		*p* < 0.001 *	
Total	64	1.00	(0.47–1.64)	0.85	(0.60–1.08)	0.33	(0.23–0.50)

* Statistically significant differences according to the Kruskal–Wallis test, for a confidence level of 95% (*p*-value < 0.05). † Statistically significant differences according to the Mann–Withney U test, for a confidence level of 95% (*p*-value < 0.05). P25, percentile 25; P75, percentile 75.

**Table 2:** Serving size, nutritional composition (energy, *trans*-fatty acids, sodium, potassium and sodium–potassium ratio) and price (per serving, per 100 g and per 100 kcal) of the street food samples collected in Dushanbe (Tajikistan), Bishkek (Kyrgyzstan), Almaty (Kazakhstan) and Chișinău (Moldova), by food group.

		Serving Size and Nutritional Composition												Food Price					
		Serving Size (g)		Energy (kcal/serving)		TFA (g/serving)		Na (mg/serving)		K (mg/serving)		Na/K		Food Price ($/serving)		Food Price ($/100 g)		Food Price ($/100 kcal)	
Tajikistan	**N**	Median (P25–P75)		Median (P25–P75)		Median (P25–P75)		Median (P25–P75)		Median (P25–P75)		Median (P25–P75)		Median (P25–P75)		Median (P25–P75)		Median (P25–P75)	
Savoury pastries	20	74	(57–93)	184	(133–225)	0.07	(0.04–0.12)	335	(248–470)	81	(71–108)	7	(6–9)	0.42	(0.42–0.42)	0.52	(0.46–0.54)	0.23	(0.16–0.28)
Sandwiches	12	217	(190–260)	447	(382–488)	0.10	(0.07–0.15)	833	(718–1237)	329	(250–409)	4	(3–6)	2.12	(1.27–2.98)	1.04	(0.68–1.07)	0.46	(0.40–0.56)
Bread	28	120	(120–120)	336	(328–364)	0.00	(0.00–0.01)	620	(522–747)	141	(126–164)	8	(6–9)	0.21	(0.21–0.21)	0.17	(0.17–0.17)	0.06	(0.06–0.06)
Snacks	4	16	(15–21)	56	(48–67)	0.11	(0.05–0.12)	431	(122–996)	36	(25–63)	18	(5–37)	0.21	(0.21–0.21)	1.18	(1.18–1.18)	0.38	(0.32–0.44)
Buns|cakes|cookies	15	73	(48–100)	244	(171–435)	0.35	(0.08–0.55)	96	(51–111)	94	(51–139)	1	(1–3)	0.85	(0.42–1.27)	0.96	(0.71–1.01)	0.25	(0.2–0.28)
Main dishes	16	274	(180–535)	477	(309–685)	0.23	(0.12–0.45)	1203	(853–2141)	581	(397–1181)	3	(2–5)	1.49	(0.84–2.98)	0.59	(0.32–0.81)	0.35	(0.17–0.41)
Ice-cream and confectionery	4	47	(43–52)	63	(56–67)	0.03	(0.03–0.06)	36	(31–63)	50	(46–63)	1	(1–2)	0.42	(0.42–0.42)	0.89	(0.89–0.89)	0.67	(0.63–0.75)
Kyrgyzstan																			
Savoury pastries	20	132	(103–151)	376	(306–437)	0.28	(0.16–0.48)	558	(402–886)	175	(125–206)	6	(5–7)	1.28	(1.28–1.28)	0.87	(0.79–0.95)	0.32	(0.26–0.38)
Sandwiches	12	234	(167–268)	527	(443–593)	0.48	(0.32–0.59)	1045	(812–1241)	405	(193–577)	5	(4–7)	2.56	(1.53–3.84)	1.11	(0.90–1.56)	0.49	(0.37–0.63)
Bread	4	120	(120–120)	335	(328–356)	0.01	(0.01–0.01)	747	(602–839)	138	(124–147)	9	(8–10)	0.25	(0.25–0.25)	0.21	(0.21–0.21)	0.07	(0.07–0.08)
Snacks	8	116	(26–278)	144	(53–268)	0.02	(0.01–0.05)	900	(164–1423)	239	(67–628)	14	(1–28)	0.89	(0.25–1.53)	0.82	(0.55–1.09)	0.50	(0.46–0.65)
Buns|cakes|cookies	12	92	(55–139)	303	(206–474)	0.13	(0.05–0.41)	194	(128–436)	102	(71–140)	4	(3–6)	1.28	(1.02–1.79)	1.54	(1.05–1.71)	0.42	(0.34–0.55)
Main dishes	20	395	(246–537)	413	(234–759)	0.14	(0.08–0.87)	1294	(827–2136)	466	(173–542)	5	(4–6)	3.07	(2.56–5.12)	0.93	(0.84–1.03)	0.78	(0.63–0.99)
Kazakhstan																			
Savoury pastries	24	107	(87–126)	321	(248–366)	0.21	(0.12–0.38)	434	(363–633)	152	(102–181)	5	(4–8)	0.79	(0.63–0.87)	0.69	(0.58–0.77)	0.22	(0.19–0.30)
Sandwiches	4	380	(354–416)	887	(789–902)	0.44	(0.31–0.58)	1745	(1550–1865)	1284	(1192–1463)	2	(2–3)	4.79	(4.79–4.79)	1.24	(1.24–1.24)	0.54	(0.53–0.62)
Bread	4	65	(53–68)	184	(153–219)	0.03	(0.02–0.39)	248	(233–289)	104	(80–109)	5	(4–6)	0.20	(0.20–0.20)	0.33	(0.33–0.33)	0.11	(0.09–0.14)
Snacks	8	96	(31–173)	137	(99–193)	0.08	(0.01–0.52)	623	(100–1121)	213	(75–420)	9	(0–25)	0.58	(0.37–0.79)	0.78	(0.36–1.19)	0.37	(0.33–0.49)
Buns|cakes|cookies	13	107	(91–136)	319	(239–416)	0.21	(0.06–1.51)	214	(94–444)	127	(69–189)	4	(2–5)	0.56	(0.47–1.19)	0.52	(0.49–1.01)	0.23	(0.16–0.32)
Main dishes	16	239	(90–441)	507	(168–737)	0.47	(0.12–0.88)	1151	(663–2034)	424	(191–756)	4	(3–7)	3.19	(1.94–3.79)	0.92	(0.68–1.69)	0.57	(0.49–0.95)
Ice-cream and confectionery	8	77	(60–134)	291	(228–298)	0.27	(0.13–1.35)	27	(12–58)	110	(69–223)	0	(0–1)	0.79	(0.47–1.11)	0.81	(0.79–0.84)	0.27	(0.16–0.49)
Moldova																			
Savoury pastries	12	112	(94–133)	334	(281–414)	0.24	(0.10–0.79)	397	(284–522)	135	(89–172)	5	(4–6)	1.26	(0.94–1.42)	1.06	(0.73–1.16)	0.38	(0.28–0.52)
Buns, cakes and cookies	12	82	(59–108)	304	(216–380)	0.47	(0.10–1.62)	153	(113–408)	47	(36–116)	5	(4–7)	0.78	(0.78–1.26)	1.24	(0.62–1.561)	0.34	(0.24–0.44)

K, potassium; Na, sodium; Na/K, sodium–potassium ratio; P25, percentile 25; P75, percentile 75; TFA, trans-fatty acids.

**Table 3:** Relation between nutrient content and food price per 100 g and per 100 kcal.

	Price per 100 g ($/100 g)				Price per 100 kcal ($/100 kcal)			
	Crude		Adjusted *		Crude		Adjusted **	
	β	95% CI	β	95% CI	β	95% CI	β	95% CI
Protein (g)	0.0014	(−0.0086, 0.0113)	−0.0035	(−0.0199,0.0130)	0.0046	(−0.0016, 0.0022)	−0.0059	(−0.0152, 0.0035)
Carbohydrates (g)	−0.0011	(−0.0045, 0.0023)	**−0.0090**	**(−0.0157, −0.0023)**	0.0003	(−0.0043, 0.0010)	**−0.0027**	**(−0.0050,−0.0004)**
Total fat (g)	**0.0106**	**(0.0011, 0.0201)**	**0.0292**	**(0.0105, 0.0479)**	0.0027	(−0.0025, 0.0079)	−0.0021	(−0.0083, 0.0040)
Monounsaturated fatty acids (g)	**0.0366**	**(0.0070, 0.0662)**	**0.0821**	**(0.0308, 0.1334)**	0.0096	(−0.0066, 0.0257)	−0.0045	(−0.0234, 0.0145)
Polyunsaturated fatty acids (g)	0.0087	(−0.0166, 0.0339)	0.0062	(−0.0282, 0.0406)	0.0056	(−0.0087, 0.0199)	−0.0058	(−0.0210, 0.095)
Saturated fatty acids (g)	**0.0218**	**(0.0016, 0.0421)**	**0.0324**	**(0.0049, 0.0600)**	0.0053	(−0.0062, 0.0168)	−0.0017	(−0.0139, 0.0105)
Trans-fatty acids (g)	**0.1504**	**(0.0156, 0.2851)**	**0.1594**	**(0.0102, 0.3086)**	−0.0016	(−0.0775, 0.0743)	−0.0147	(−0.0888, 0.0594)
Sodium (g)	−0.0319	(−0.1915, 0.1278)	−0.1150	(−0.3337, 0.1037)	**0.1209**	**(0.0369, 0.2048)**	0.0369	(−0.1215, 0.1952)
Potassium (g)	0.0340	(−0.2772, 0.3452)	−0.1088	(−0.5275, 0.3099)	**0.2127**	**(0.0529, 0.3725)**	0.0851	(−0.1356, 0.3059)

* Adjusted for energy (kcal). ** Adjusted for serving size (g). CI, confidence interval. Significant results are presented in bold.
